# Supplementary figures and images for: Assessing alignment-based taxonomic classification of ancient microbial DNA
Source: PeerJ. 2019 Mar 13;7:e6594. doi: 10.7717/peerj.6594 (PMC6420809; doi:10.7717/peerj.6594)

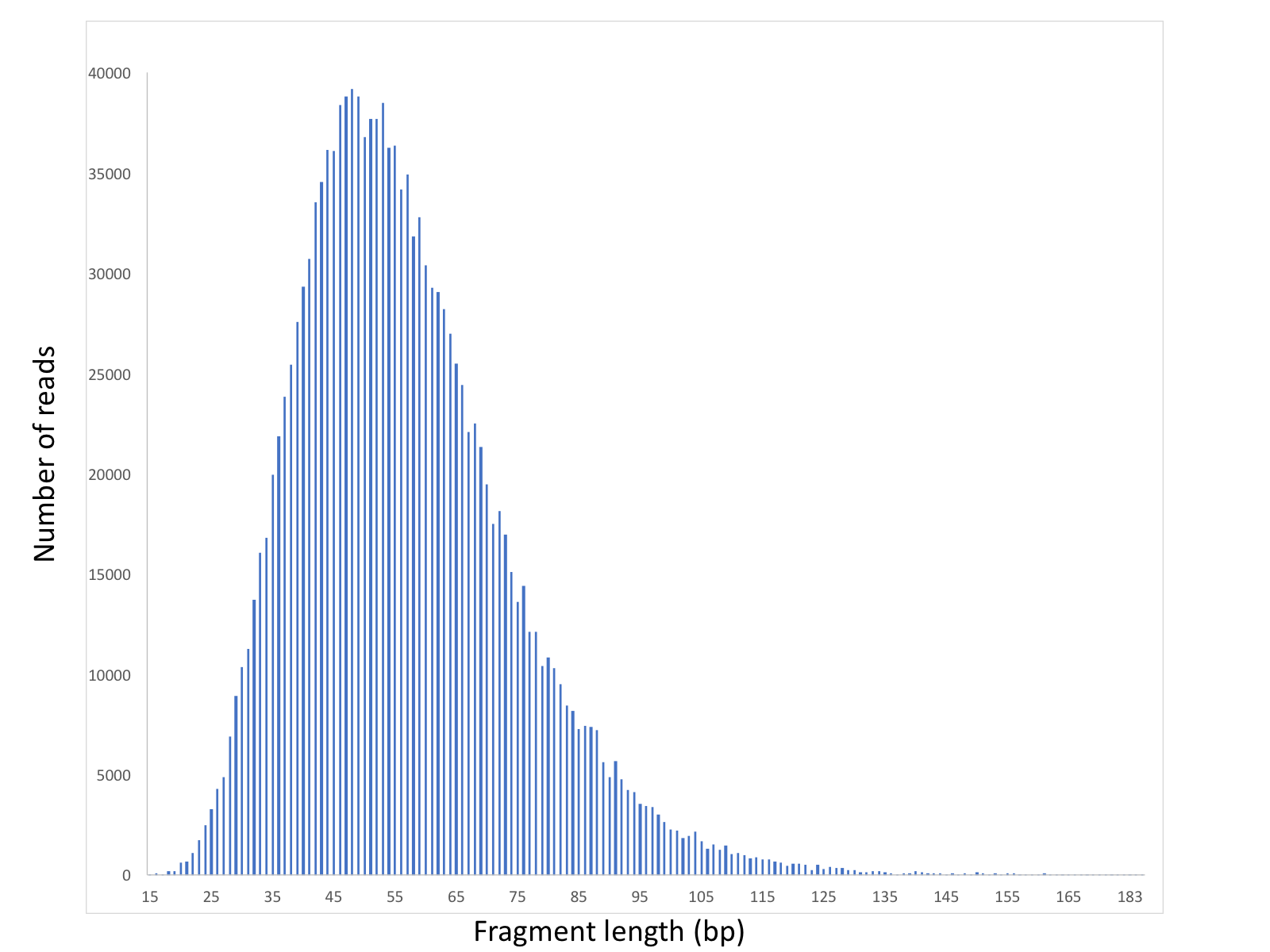

Supplement: Supplemental Information 1 [file peerj-07-6594-s001.png]

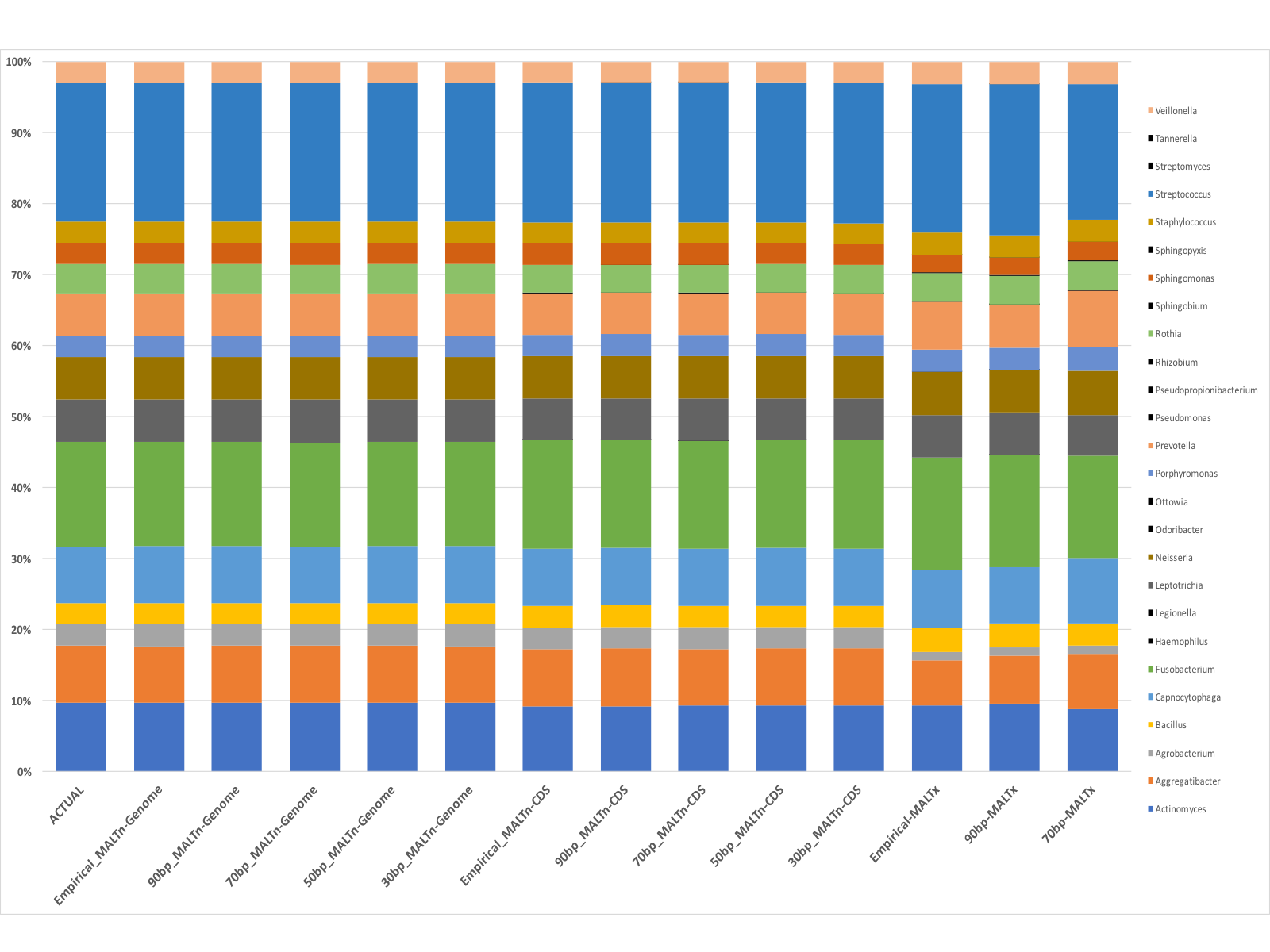

Supplement: Supplemental Information 2 — Taxa coloured black were not used as input for constructing the simulated metagenomes and represent misclassifications. [file peerj-07-6594-s002.png]

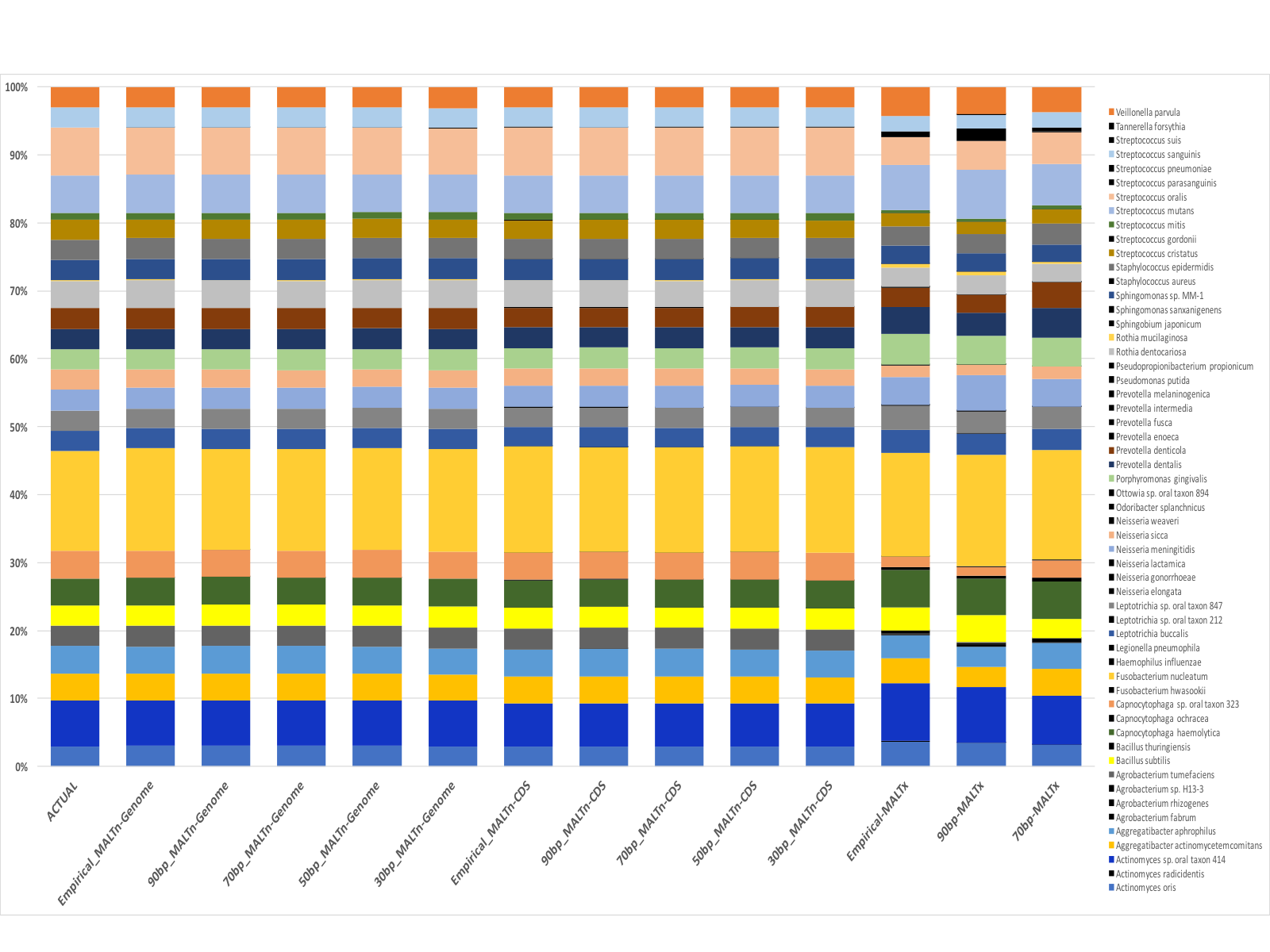

Supplement: Supplemental Information 3 — Taxa coloured black were not used as input for constructing the simulated metagenomes and represent misclassifications. [file peerj-07-6594-s003.png]

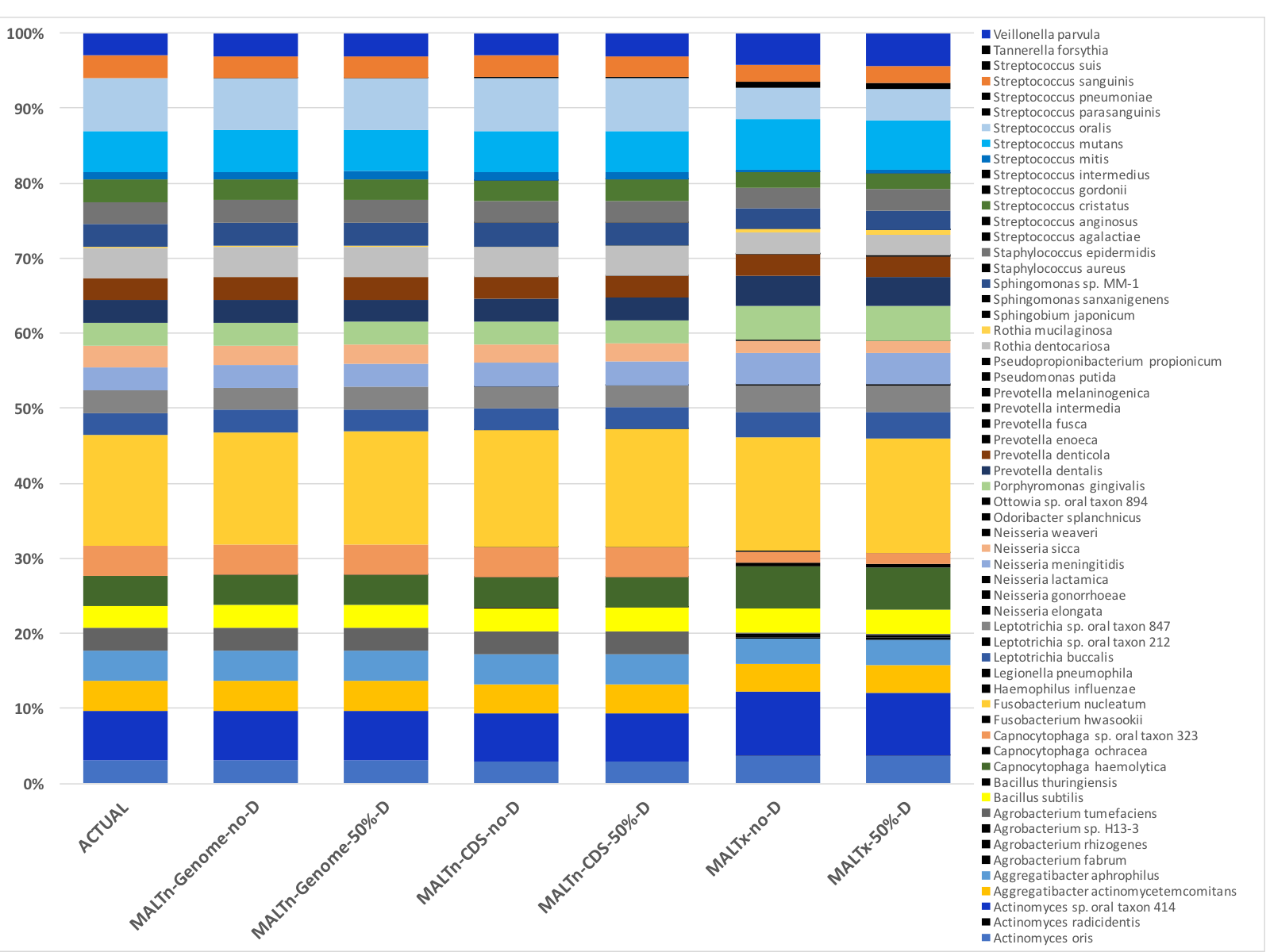

Supplement: Supplemental Information 4 — Taxa coloured black were not used as input for constructing the simulated metagenomes and represent misclassifications. [file peerj-07-6594-s004.png]

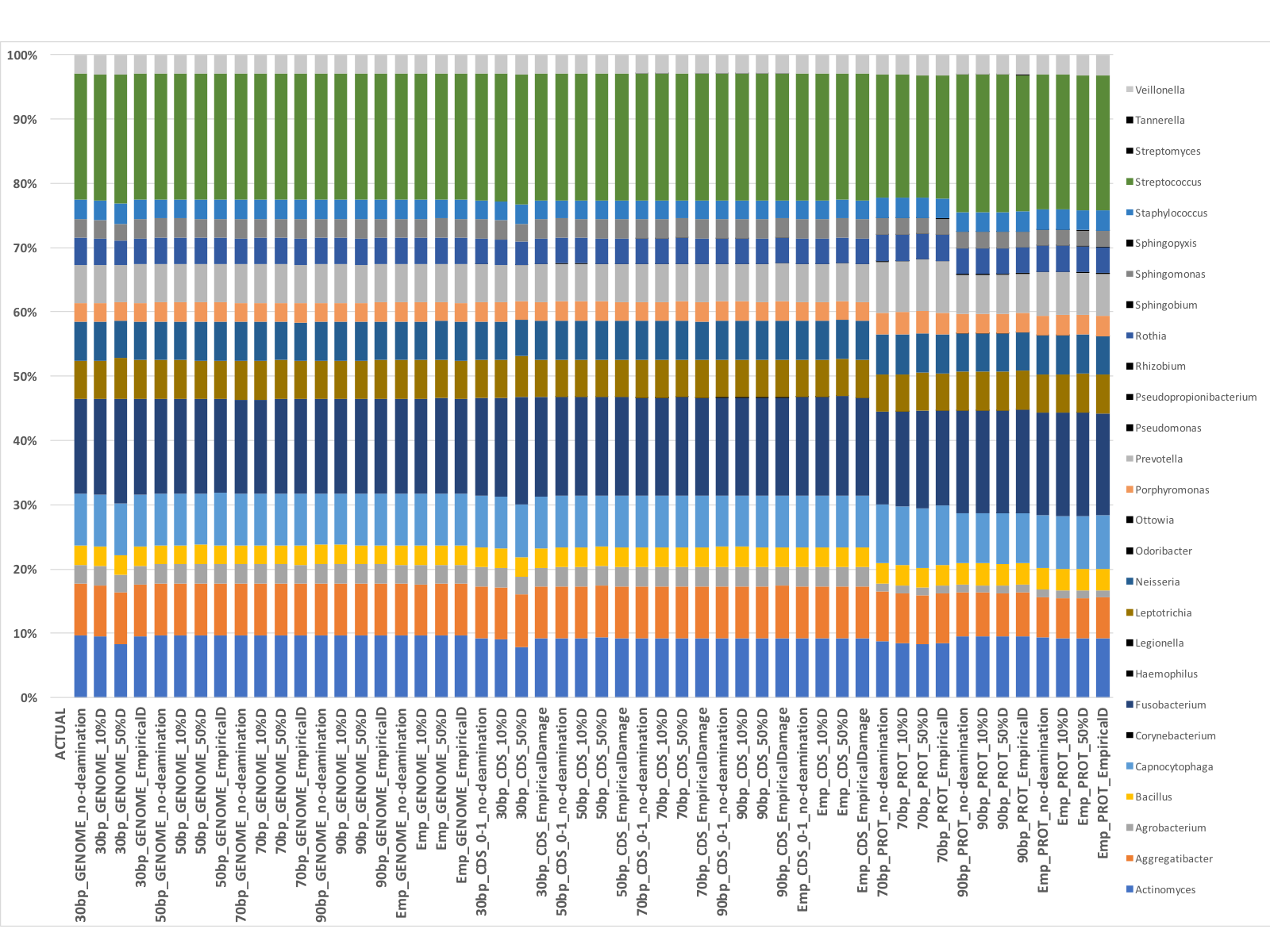

Supplement: Supplemental Information 5 — Taxa coloured black were not used as input for constructing the simulated metagenomes and represent misclassifications. [file peerj-07-6594-s005.png]

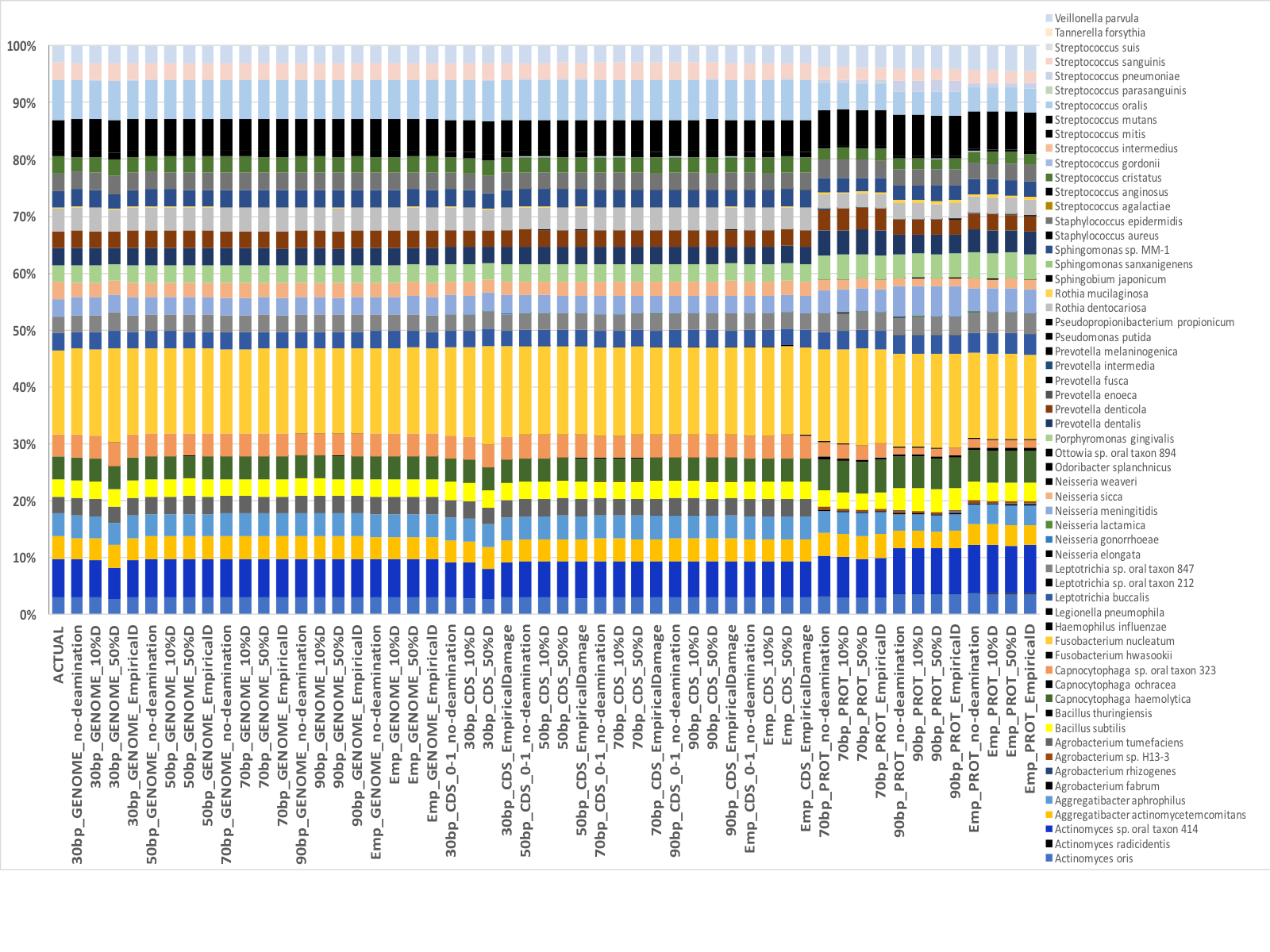

Supplement: Supplemental Information 6 — Taxa coloured black were not used as input for constructing the simulated metagenomes and represent misclassifications. [file peerj-07-6594-s006.png]

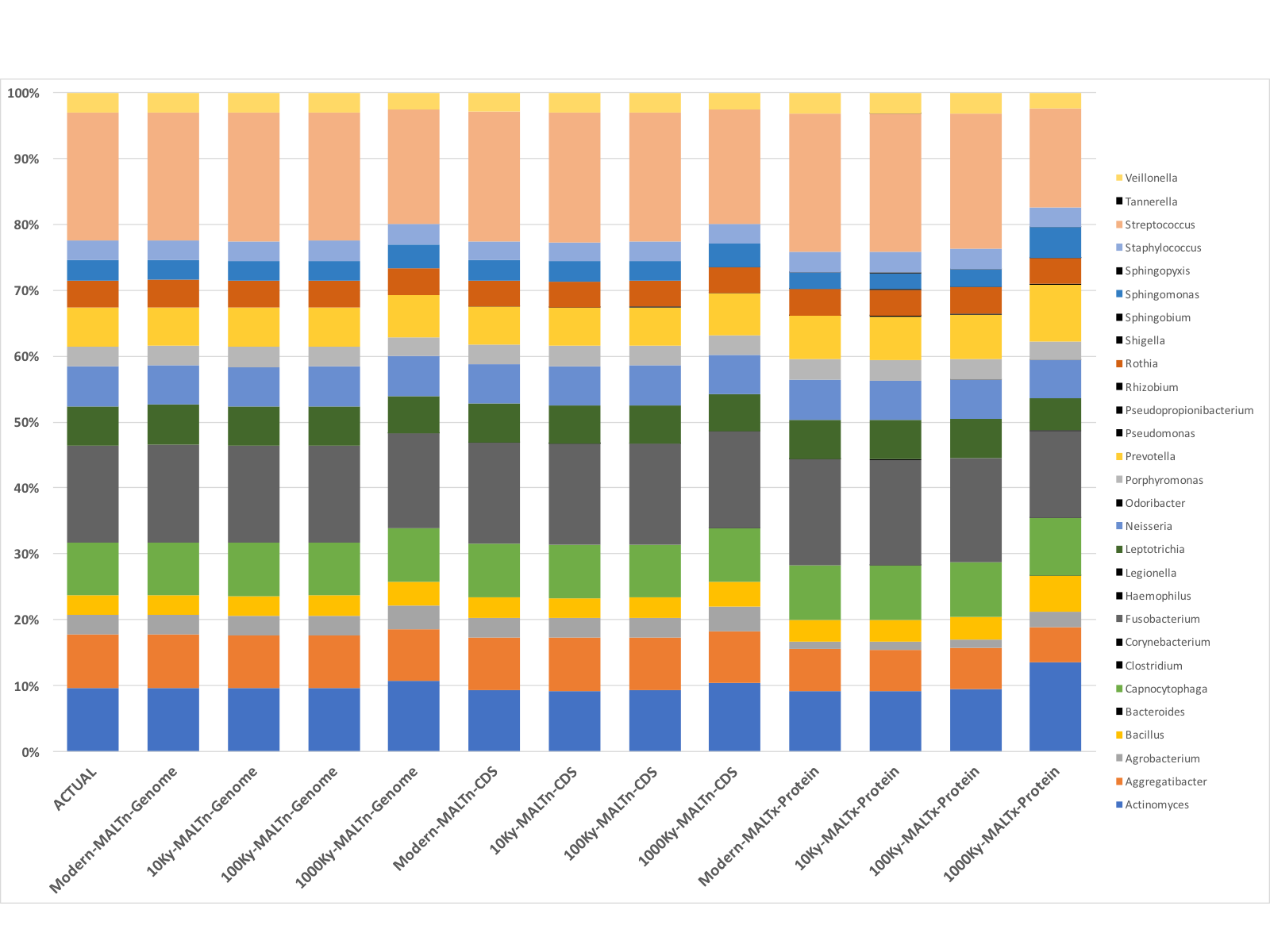

Supplement: Supplemental Information 7 — Taxa coloured black were not used as input for constructing the simulated metagenomes and represent misclassifications. [file peerj-07-6594-s007.png]

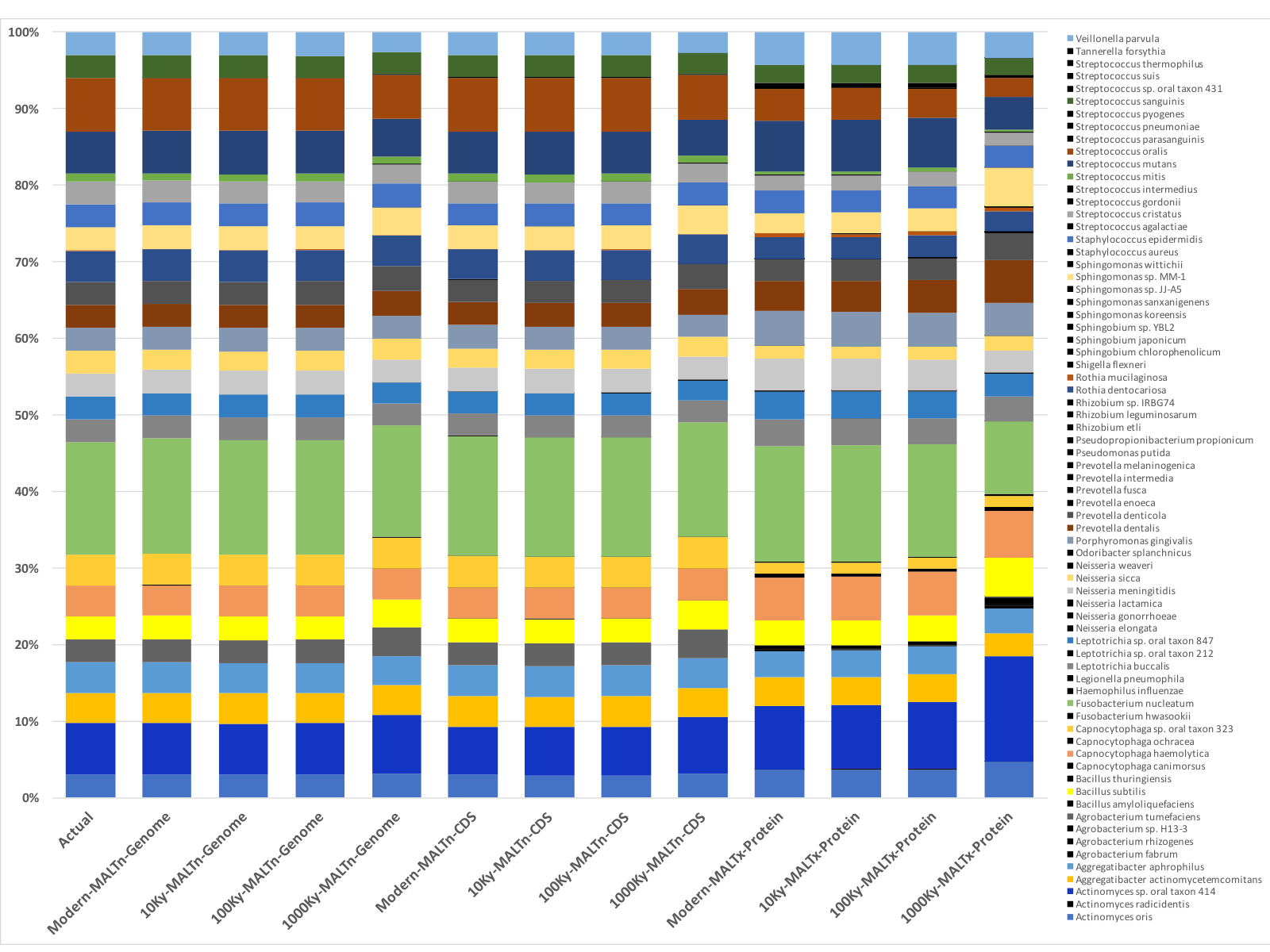

Supplement: Supplemental Information 8 — Taxa coloured black were not used as input for constructing the simulated metagenomes and represent misclassifications. [file peerj-07-6594-s008.png]

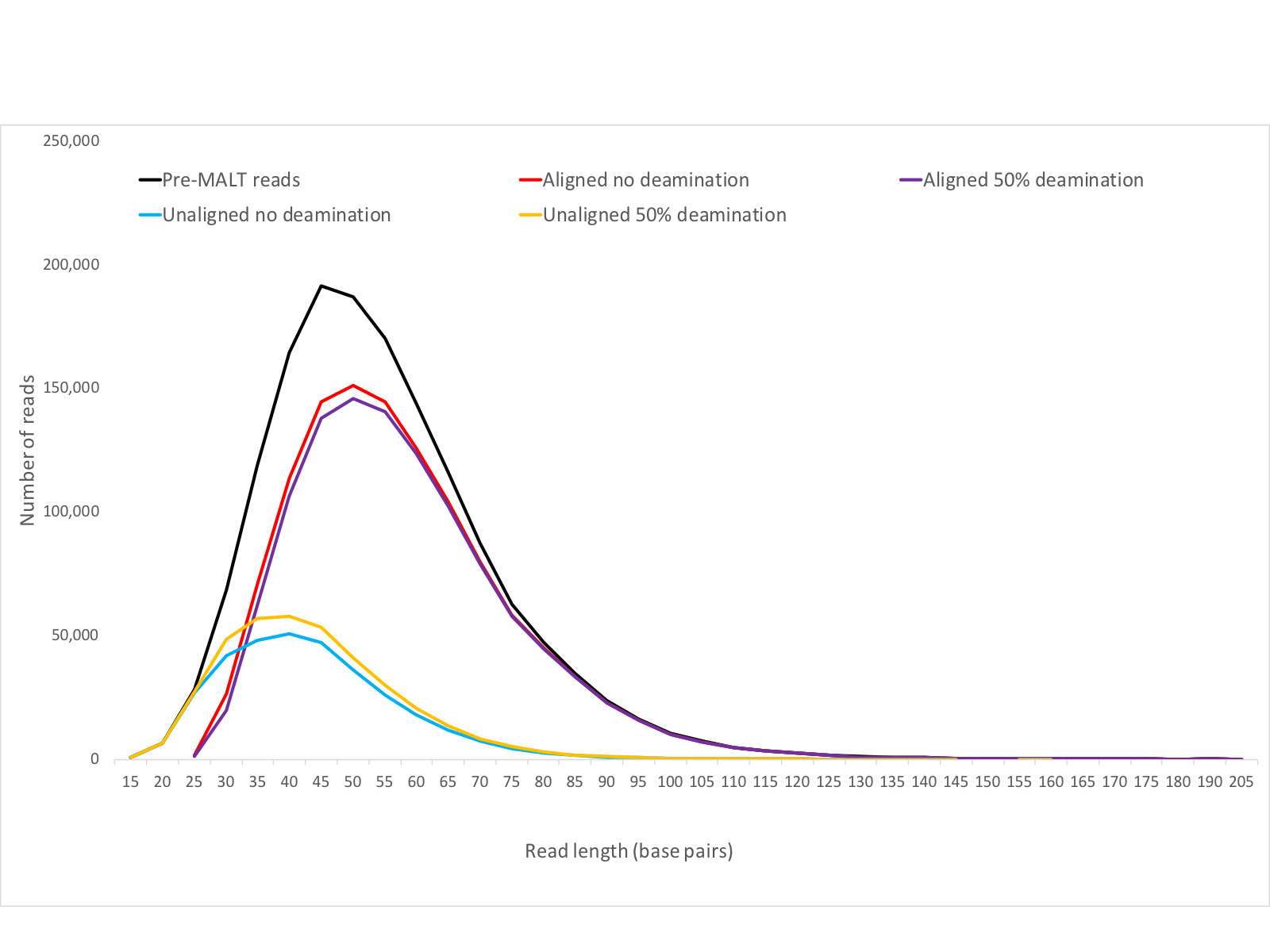

Supplement: Supplemental Information 9 [file peerj-07-6594-s009.png]

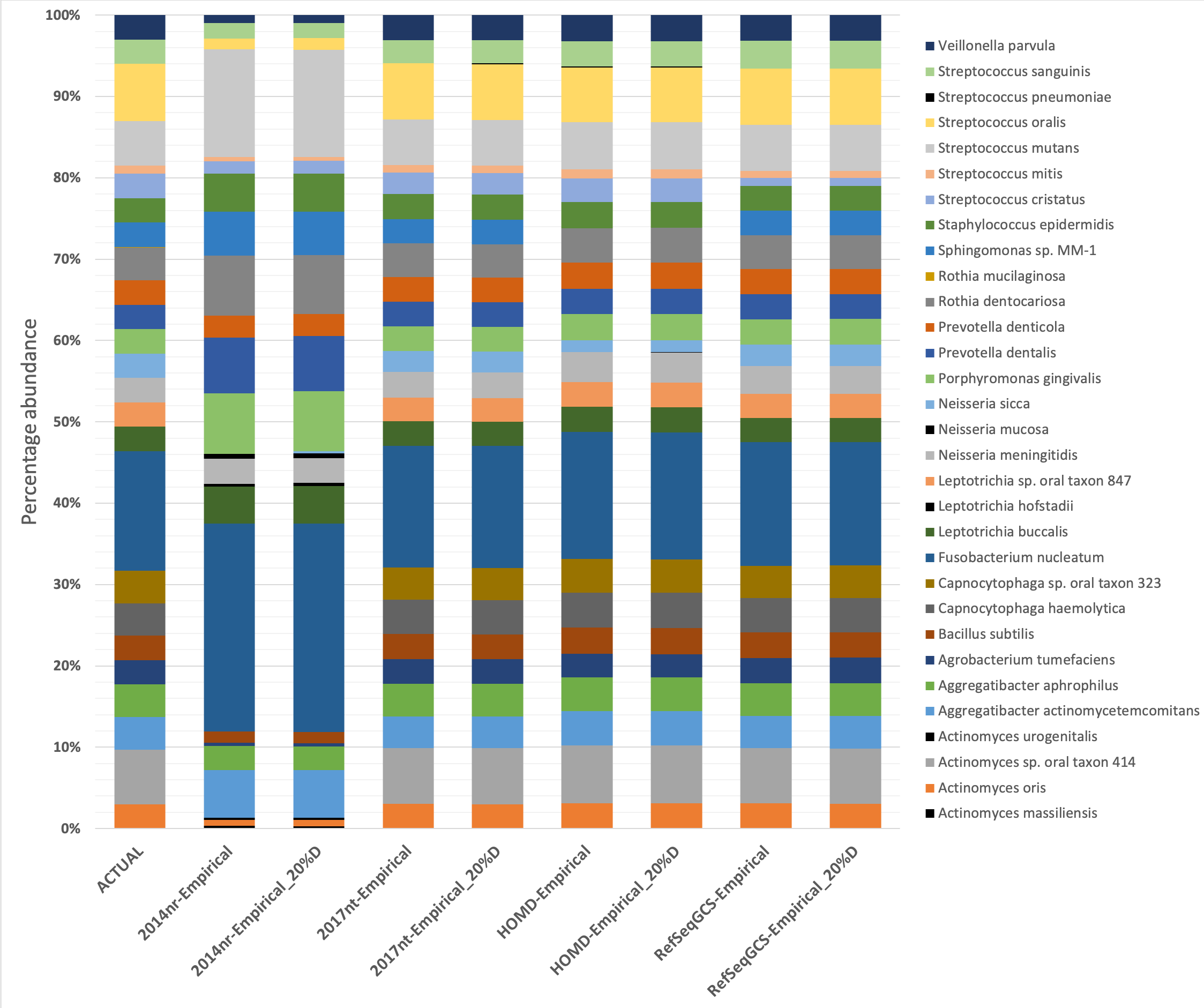

Supplement: Supplemental Information 10 — The empirical read length distribution simulated metagenome with moderate (20%D) or no deamination (Metagenomes 17 and 20 from table S2) were used as input against four different nucleotide MALT databases. Taxa coloured black represent false positive assignments. [file peerj-07-6594-s010.png]

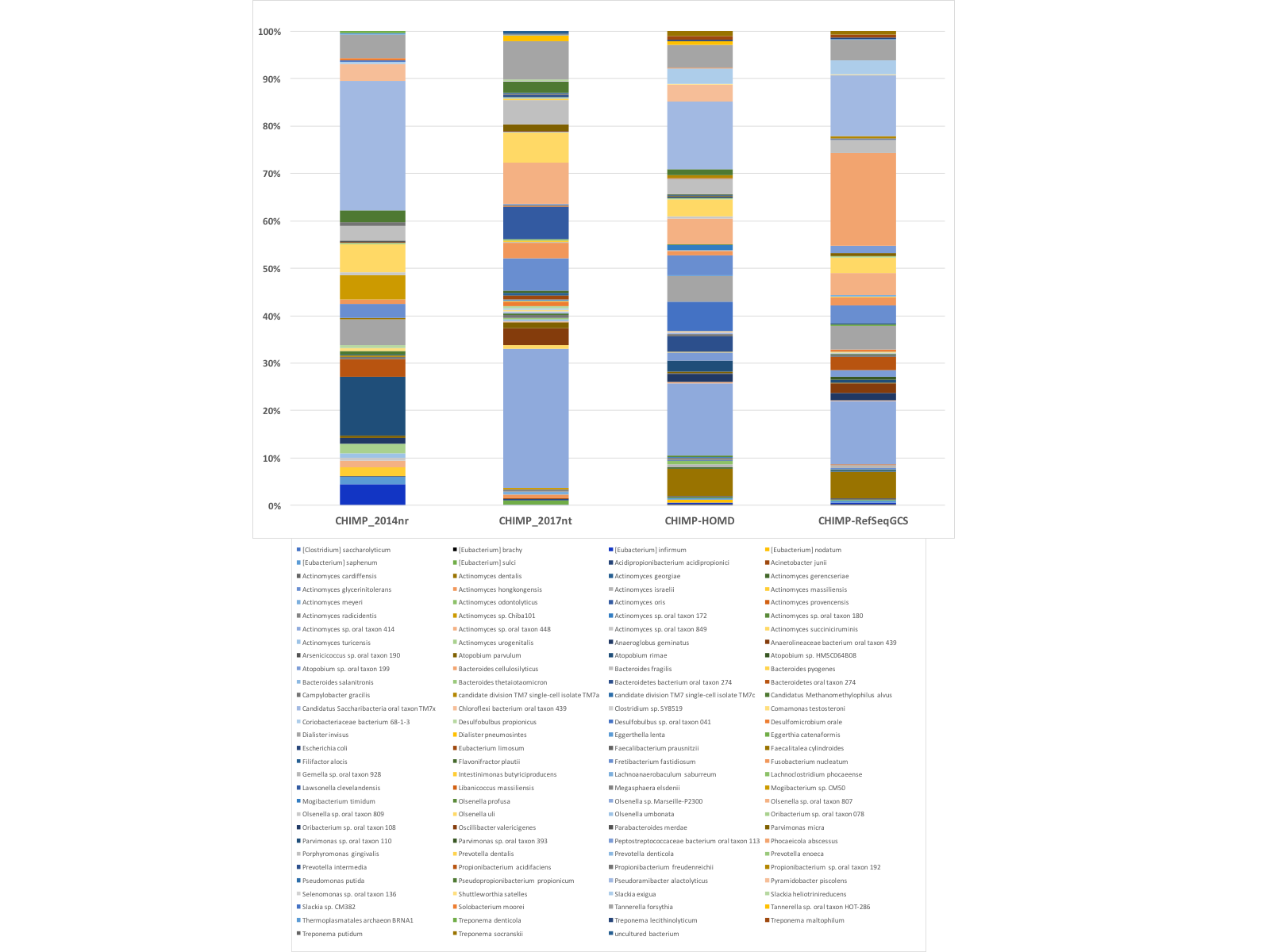

Supplement: Supplemental Information 11 — Species-level classification of the Chimpanzee dental calculus sample from Weyrich et al 2017 using different MALT databases. [file peerj-07-6594-s011.png]

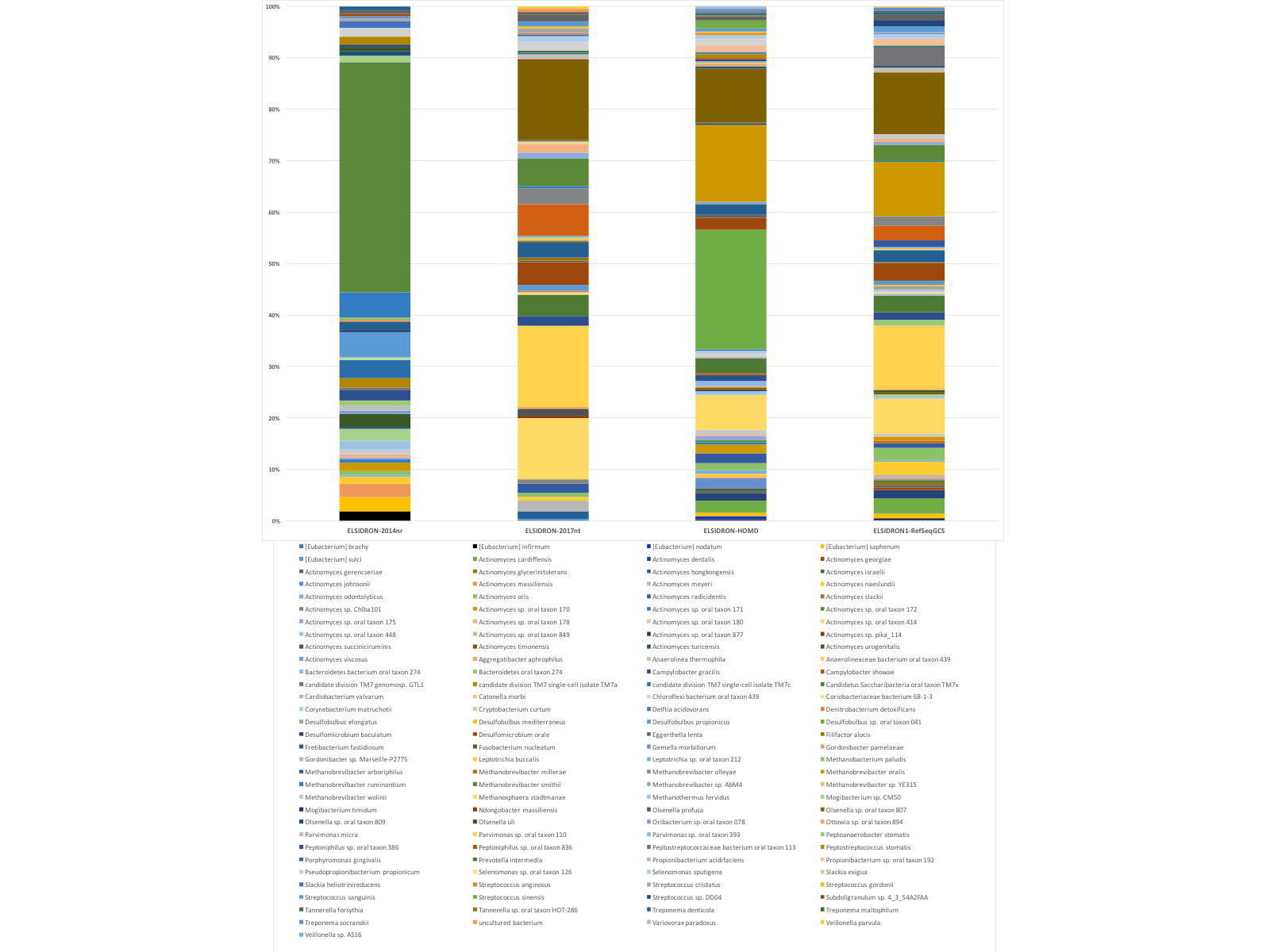

Supplement: Supplemental Information 12 — Species-level classification of the El Sidron1 Neanderthal dental calculus sample from Weyrich et al 2017 using different MALT database. [file peerj-07-6594-s012.png]

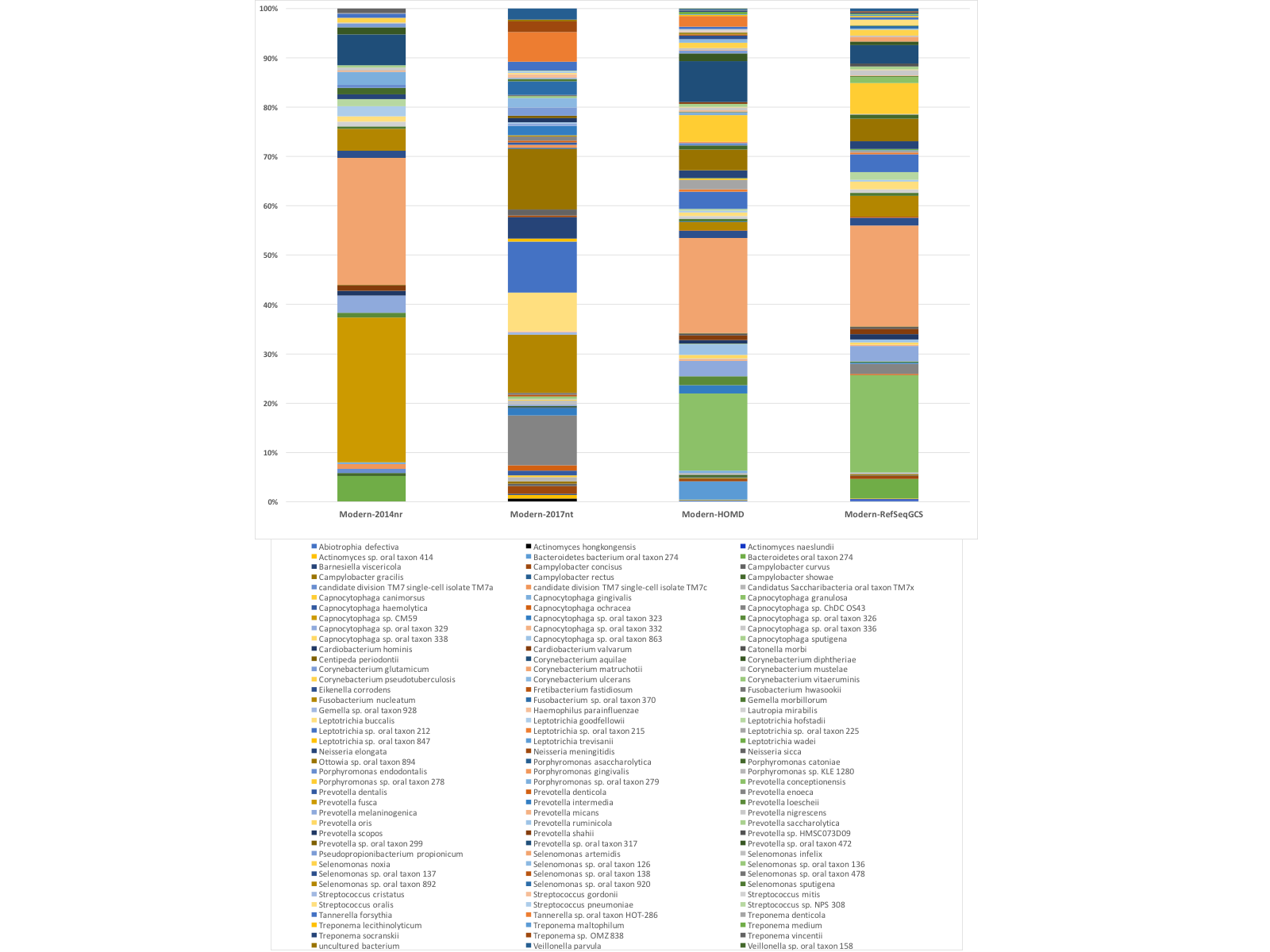

Supplement: Supplemental Information 13 — Species-level classification of the modern dental calculus sample from Weyrich et al 2017 using different MALT databases. [file peerj-07-6594-s013.png]

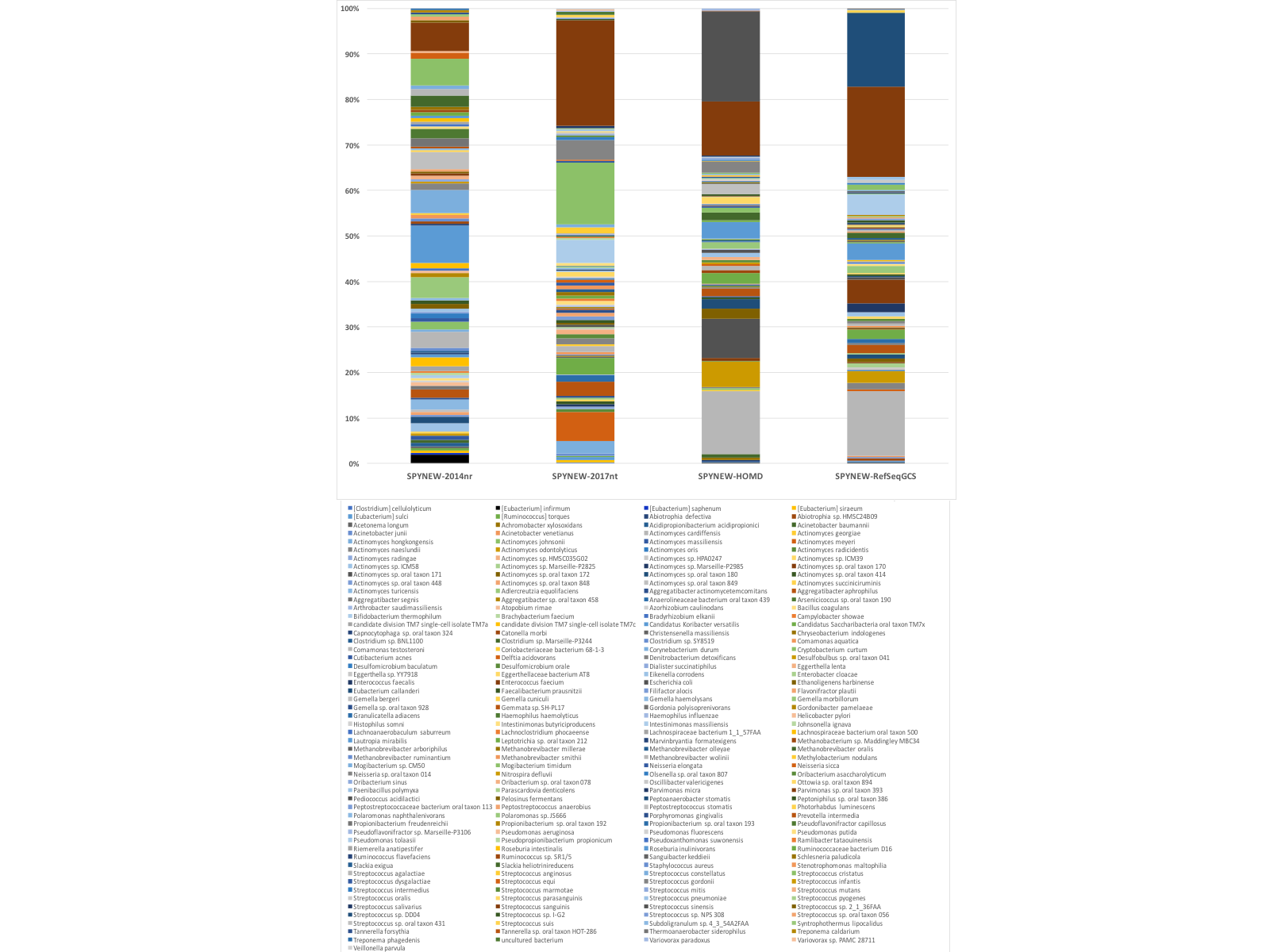

Supplement: Supplemental Information 14 — Species-level classification of the Spy II Neanderthal dental calculus sample from Weyrich et al 2017 using different MALT databases. [file peerj-07-6594-s014.png]

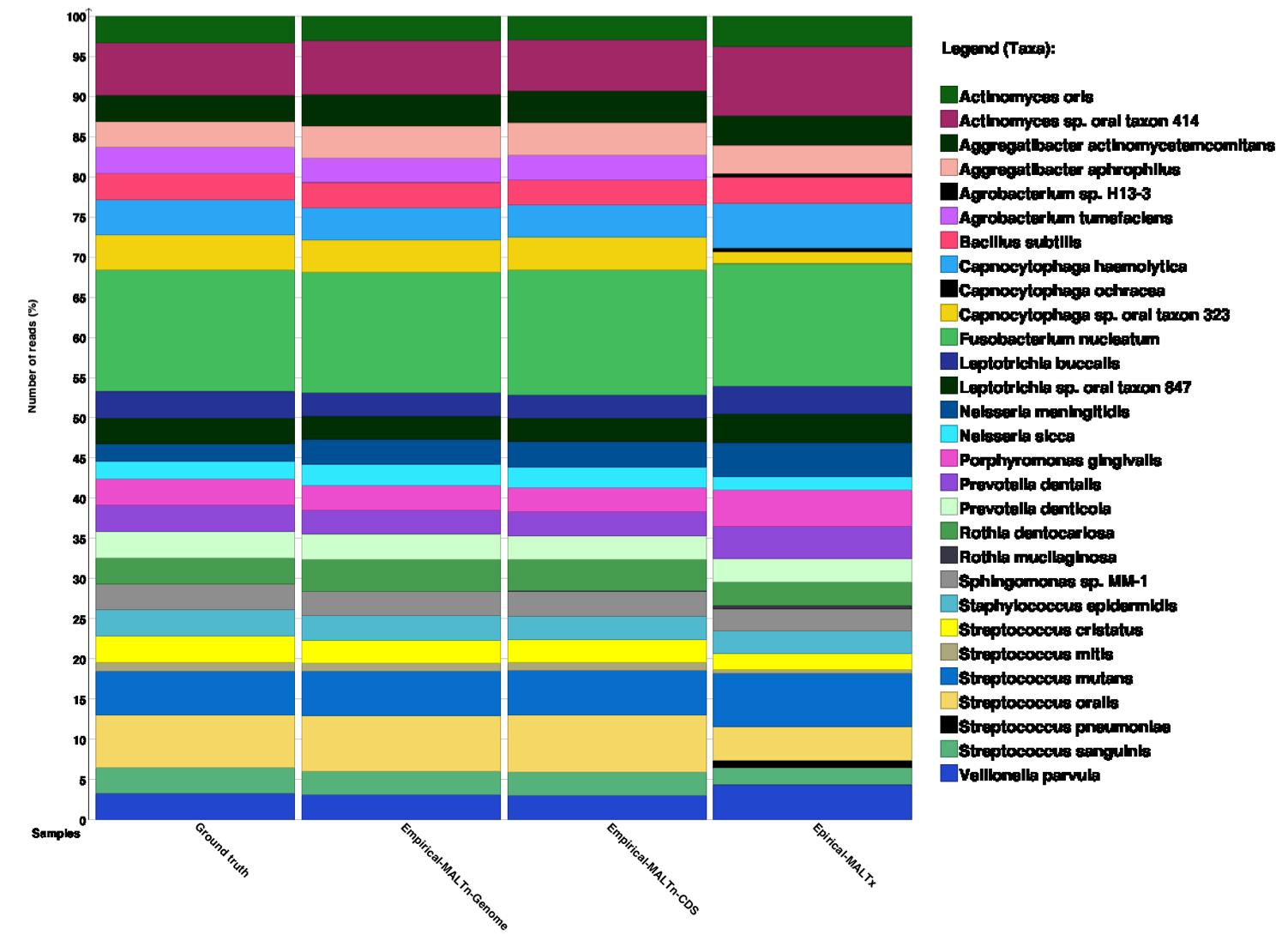

Supplement: Supplemental Information 15 — The empirical ancient DNA fragment length distribution simulated metagenome without deamination was used as input. Taxa coloured black represent false positive assignments. [file peerj-07-6594-s015.png]
