# Supplementary material for: Assessing alignment-based taxonomic classification of ancient microbial DNA
Source: PeerJ. 2019 Mar 13;7:e6594. doi: 10.7717/peerj.6594 (PMC6420809; doi:10.7717/peerj.6594)
Supplement: Supplemental Information 16 — Plaque community based on Mark-Welsh et al. 2016. [file peerj-07-6594-s016.docx]

| **Taxon** | **fna filename:** | **Abundance** | **% Abundance** | **Genus** | **% Abundance Genus** |
| --- | --- | --- | --- | --- | --- |
| Actinomyces oris strain T14V | GCF_001553935.1_ASM155393v1_cds_from_genomic.fna | 0.03 | 3 | Actinomyces |  |
| Actinomyces sp. oral taxon 414 strain F0588 | GCF_001278845.1_ASM127884v1_cds_from_genomic.fna | 0.067 | 6.7 | Actinomyces | 9.7 |
| Aggregatibacter actinomycetemcomitans strain 624 | GCF_001594265.1_ASM159426v1_cds_from_genomic.fna | 0.04 | 4 | Aggregatibacter |  |
| Aggregatibacter aphrophilus strain W10433 | GCF_001262035.1_ASM126203v1_cds_from_genomic.fna | 0.04 | 4 | Aggregatibacter | 8 |
| Agrobacterium tumefaciens strain A | GCF_000971565.1_ASM97156v1_cds_from_genomic.fna | 0.03 | 3 | Agrobacterium | 3 |
| Bacillus subtilis BSn5 | GCF_000186745.1_ASM18674v1_cds_from_genomic.fna | 0.03 | 3 | Bacillus | 3 |
| Capnocytophaga haemolytica strain CCUG 32990 | GCF_001553545.1_ASM155354v1_cds_from_genomic.fna | 0.04 | 4 | Capnocytophaga |  |
| Capnocytophaga sp. oral taxon 323 strain F0383 | GCF_001278825.1_ASM127882v1_cds_from_genomic.fna | 0.04 | 4 | Capnocytophaga | 8 |
| Fusobacterium nucleatum subsp. nucleatum ATCC 25586 | GCF_000007325.1_ASM732v1_cds_from_genomic.fna | 0.1 | 10 | Fusobacterium |  |
| Fusobacterium nucleatum subsp. polymorphum strain ChDC F306 | GCF_001433955.1_ASM143395v1_cds_from_genomic.fna | 0.04 | 4 | Fusobacterium |  |
| Fusobacterium nucleatum subsp. vincentii 3_1_36A2 | GCF_000162235.2_ASM16223v2_cds_from_genomic.fna | 0.007 | 0.7 | Fusobacterium | 14.7 |
| Leptotrichia buccalis DSM 113 | GCF_000023905.1_ASM2390v1_cds_from_genomic.fna | 0.03 | 3 | Leptotrichia |  |
| Leptotrichia sp. oral taxon 847 | GCF_001553645.1_ASM155364v1_cds_from_genomic.fna | 0.03 | 3 | Leptotrichia | 6 |
| Neisseria meningitidis MC58 chromosome | GCF_000008805.1_ASM880v1_cds_from_genomic.fna | 0.03 | 3 | Neisseria |  |
| Neisseria sicca strain FDAARGOS_2 | GCF_002073715.1_ASM207371v1_cds_from_genomic.fna | 0.03 | 3 | Neisseria | 6 |
| Porphyromonas gingivalis ATCC 33277 DNA | GCF_000010505.1_ASM1050v1_cds_from_genomic.fna | 0.03 | 3 | Porphyromonas | 3 |
| Prevotella dentalis DSM 3688 | GCF_000242335.1_ASM24233v3_cds_from_genomic.fna | 0.03 | 3 | Prevotella |  |
| Prevotella denticola F0289 | GCF_000193395.1_ASM19339v1_cds_from_genomic.fna | 0.03 | 3 | Prevotella | 6 |
| Rothia dentocariosa ATCC 17931 | GCF_000164695.2_ASM16469v2_cds_from_genomic.fna | 0.04 | 4 | Rothia |  |
| Rothia mucilaginosa DNA complete genome strain: NUM-Rm6536 | GCF_001548235.1_ASM154823v1_cds_from_genomic.fna | 0.001 | 0.1 | Rothia | 4.1 |
| Sphingomonas sp. MM-1 | GCF_000347675.2_ASM34767v2_cds_from_genomic.fna | 0.03 | 3 | Sphingomonas | 3 |
| Staphylococcus epidermidis ATCC 12228 | GCF_000007645.1_ASM764v1_cds_from_genomic.fna | 0.03 | 3 | Staphylococcus | 3 |
| Streptococcus cristatus AS 1.3089 | GCF_000385925.1_ASM38592v1_cds_from_genomic.fna | 0.03 | 3 | Streptococcus |  |
| Streptococcus mitis B6 | GCF_000027165.1_ASM2716v1_cds_from_genomic.fna | 0.01 | 1 | Streptococcus |  |
| Streptococcus mutans NN202DNA | GCF_000091645.1_ASM9164v1_cds_from_genomic.fna | 0.005 | 0.5 | Streptococcus |  |
| Streptococcus mutans UA159 chromosome | GCF_000007465.2_ASM746v2_cds_from_genomic.fna | 0.05 | 5 | Streptococcus |  |
| Streptococcus oralis Uo5 | GCF_000253155.1_ASM25315v1_cds_from_genomic.fna | 0.07 | 7 | Streptococcus |  |
| Streptococcus sanguinis SK36 | GCF_000014205.1_ASM1420v1_cds_from_genomic.fna | 0.03 | 3 | Streptococcus | 19.5 |
| Veillonella parvula DSM 2008 | GCF_000024945.1_ASM2494v1_cds_from_genomic.fna | 0.03 | 3 | Veillonella | 3 |
|  | **TOTAL:** | **1** | **100** |  | **100** |
