# Supplementary material for: Assessing alignment-based taxonomic classification of ancient microbial DNA
Source: PeerJ. 2019 Mar 13;7:e6594. doi: 10.7717/peerj.6594 (PMC6420809; doi:10.7717/peerj.6594)
Supplement: Supplemental Information 17 [file peerj-07-6594-s017.docx]

|  | **Fragment length (BP)** | **Deamination (%ss)** | **Divergence (% nucleotides)** |
| --- | --- | --- | --- |
| Metagenome1 | 30 | 0 | 0 |
| Metagenome2 | 30 | 0.1 | 0 |
| Metagenome3 | 30 | 0.5 | 0 |
| Metagenome4 | 30 | Empirical | 0 |
| Metagenome5 | 50 | 0 | 0 |
| Metagenome6 | 50 | 0.1 | 0 |
| Metagenome7 | 50 | 0.5 | 0 |
| Metagenome8 | 50 | Empirical | 0 |
| Metagenome9 | 70 | 0 | 0 |
| Metagenome10 | 70 | 0.1 | 0 |
| Metagenome11 | 70 | 0.5 | 0 |
| Metagenome12 | 70 | Empirical | 0 |
| Metagenome13 | 90 | 0 | 0 |
| Metagenome14 | 90 | 0.1 | 0 |
| Metagenome15 | 90 | 0.5 | 0 |
| Metagenome16 | 90 | Empirical | 0 |
| Metagenome17 | Empirical | 0 | 0 |
| Metagenome18 | Empirical | 0.1 | 0 |
| Metagenome19 | Empirical | 0.5 | 0 |
| Metagenome20 | Empirical | Empirical | 0 |
| Metagenome21 | Empirical | 0.5 | 0.1 |
| Metagenome22 | Empirical | 0.5 | 1 |
| Metagenome23 | Empirical | 0.5 | 10 |
