# Supplementary material for: Assessing alignment-based taxonomic classification of ancient microbial DNA
Source: PeerJ. 2019 Mar 13;7:e6594. doi: 10.7717/peerj.6594 (PMC6420809; doi:10.7717/peerj.6594)
Supplement: Supplemental Information 18 [file peerj-07-6594-s018.docx]

| **MALTx** | **MALTn-CDS** | **MALTn-Genome** |
| --- | --- | --- |
| Actinomyces radicidentis | Agrobacterium fabrum | Capnocytophaga ochracea |
| Agrobacterium fabrum | Bacillus thuringiensis | Streptococcus parasanguinis |
| Agrobacterium rhizogenes | Capnocytophaga ochracea |  |
| Agrobacterium sp. H13-3 | Fusobacterium hwasookii |  |
| Capnocytophaga ochracea | Haemophilus influenzae |  |
| Fusobacterium hwasookii | Legionella pneumophila |  |
| Leptotrichia sp. oral taxon 212 | Neisseria gonorrhoeae |  |
| Neisseria elongata | Neisseria lactamica |  |
| Neisseria gonorrhoeae | Pseudomonas putida |  |
| Neisseria lactamica | Staphylococcus aureus |  |
| Neisseria weaveri | Streptococcus gordonii |  |
| Odoribacter splanchnicus |  |  |
| Ottowia sp. oral taxon 894 |  |  |
| Prevotella enoeca |  |  |
| Prevotella fusca |  |  |
| Prevotella intermedia |  |  |
| Prevotella melaninogenica |  |  |
| Pseudopropionibacterium propionicum |  |  |
| Sphingobium japonicum |  |  |
| Sphingomonas sanxanigenens |  |  |
| Streptococcus gordonii |  |  |
| Streptococcus pneumoniae |  |  |
| Streptococcus suis |  |  |
| Tannerella forsythia |  |  |
