# Supplementary material for: Assessing alignment-based taxonomic classification of ancient microbial DNA
Source: PeerJ. 2019 Mar 13;7:e6594. doi: 10.7717/peerj.6594 (PMC6420809; doi:10.7717/peerj.6594)
Supplement: Supplemental Information 19 [file peerj-07-6594-s019.docx]

| **Fragment length** | **Reads assigned total** | **Reads assigned genus** | **Reads assigned species** |
| --- | --- | --- | --- |
| 30bp_MALTn-genome_0%D | 99.97% | 96.04% | 74.55% |
| 30bp_MALTn-genome_10%D | 98.11% | 94.65% | 74.37% |
| 30bp_MALTn-genome_50%D | 84.21% | 81.55% | 65.02% |
| 30bp_MALTn-genome_20% | 98.54% | 95.06% | 74.78% |
| 50bp_MALTn-genome_0%D | 99.92% | 97.93% | 77.97% |
| 50bp_MALTn-genome_10%D | 99.94% | 98.00% | 78.51% |
| 50bp_MALTn-genome_50%D | 99.93% | 98.03% | 78.98% |
| 50bp_MALTn-genome_20% | 99.93% | 98.02% | 78.57% |
| 70bp_MALTn-genome_0%D | 99.95% | 98.63% | 82.77% |
| 70bp_MALTn-genome_10%D | 99.96% | 98.63% | 82.64% |
| 70bp_MALTn-genome_50%D | 99.97% | 98.61% | 82.32% |
| 70bp_MALTn-genome_20% | 99.97% | 98.64% | 82.58% |
| 90bp_MALTn-genome_0%D | 99.97% | 98.75% | 82.89% |
| 90bp_MALTn-genome_10%D | 99.98% | 98.74% | 82.80% |
| 90bp_MALTn-genome_50%D | 99.99% | 98.74% | 82.68% |
| 90bp_MALTn-genome_20% | 99.98% | 98.77% | 82.93% |
| Emp_MALTn-genome_0%D | 98.62% | 96.91% | 79.30% |
| Emp_MALTn-genome_10%D | 98.44% | 96.70% | 79.11% |
| Emp_MALTn-genome_50%D | 97.72% | 95.93% | 78.33% |
| Emp_MALTn-genome_20% | 98.48% | 96.72% | 79.05% |
