# Supplementary material for: Assessing alignment-based taxonomic classification of ancient microbial DNA
Source: PeerJ. 2019 Mar 13;7:e6594. doi: 10.7717/peerj.6594 (PMC6420809; doi:10.7717/peerj.6594)
Supplement: Supplemental Information 20 [file peerj-07-6594-s020.docx]

| **Fragment length** | **Reads assigned total** | **Reads assigned genus** | **Reads assigned species** |
| --- | --- | --- | --- |
| 30bp_MALTn-CDS_0%D | 85.80% | 82.66% | 63.70% |
| 30bp_MALTn-CDS_10%D | 84.19% | 81.36% | 63.52% |
| 30bp_MALTn-CDS_50%D | 71.81% | 69.70% | 55.29% |
| 30bp_MALTn-CDS_20% | 84.57% | 81.74% | 63.90% |
| 50bp_MALTn-CDS_0%D | 88.07% | 86.48% | 68.38% |
| 50bp_MALTn-CDS_10%D | 88.01% | 86.48% | 68.83% |
| 50bp_MALTn-CDS_50%D | 87.96% | 86.45% | 69.23% |
| 50bp_MALTn-CDS_20% | 88.02% | 86.48% | 68.88% |
| 70bp_MALTn-CDS_0%D | 89.84% | 88.67% | 73.88% |
| 70bp_MALTn-CDS_10%D | 89.79% | 88.62% | 73.73% |
| 70bp_MALTn-CDS_50%D | 89.78% | 88.60% | 73.45% |
| 70bp_MALTn-CDS_20% | 89.77% | 88.60% | 73.66% |
| 90bp_MALTn-CDS_0%D | 91.26% | 90.07% | 75.02% |
| 90bp_MALTn-CDS_10%D | 91.22% | 90.03% | 74.90% |
| 90bp_MALTn-CDS_50%D | 91.12% | 89.93% | 74.71% |
| 90bp_MALTn-CDS_20% | 91.18% | 90.00% | 75.01% |
| Emp_MALTn-CDS_0%D | 87.44% | 86.01% | 69.95% |
| Emp_MALTn-CDS_10%D | 87.22% | 85.78% | 69.75% |
| Emp_MALTn-CDS_50%D | 86.52% | 85.04% | 69.01% |
| Emp_MALTn-CDS_20% | 87.21% | 85.75% | 69.66% |
