# Supplementary material for: Assessing alignment-based taxonomic classification of ancient microbial DNA
Source: PeerJ. 2019 Mar 13;7:e6594. doi: 10.7717/peerj.6594 (PMC6420809; doi:10.7717/peerj.6594)
Supplement: Supplemental Information 21 [file peerj-07-6594-s021.docx]

| **Fragment length** | **Reads assigned total** | **Reads assigned genus** | **Reads assigned species** |
| --- | --- | --- | --- |
| 30bp_MALTx_0%D | 0.00% | 0.00% | 0.00% |
| 30bp_MALTx_10%D | 0.00% | 0.00% | 0.00% |
| 30bp_MALTx_50%D | 0.00% | 0.00% | 0.00% |
| 30bp_MALTx_20% | 0.00% | 0.00% | 0.00% |
| 50bp_MALTx_0%D | 0.00% | 0.00% | 0.00% |
| 50bp_MALTx_10%D | 0.00% | 0.00% | 0.00% |
| 50bp_MALTx_50%D | 0.00% | 0.00% | 0.00% |
| 50bp_MALTx_20% | 0.00% | 0.00% | 0.00% |
| 70bp_MALTx_0%D | 33.02% | 29.83% | 20.51% |
| 70bp_MALTx_10%D | 29.94% | 27.08% | 18.64% |
| 70bp_MALTx_50%D | 26.04% | 23.58% | 16.24% |
| 70bp_MALTx_20% | 29.21% | 26.40% | 18.22% |
| 90bp_MALTx_0%D | 82.17% | 70.68% | 40.14% |
| 90bp_MALTx_10%D | 81.82% | 70.34% | 39.94% |
| 90bp_MALTx_50%D | 81.70% | 70.21% | 39.86% |
| 90bp_MALTx_20% | 81.51% | 70.09% | 39.80% |
| Emp_MALTx_0%D | 15.77% | 13.84% | 8.46% |
| Emp_MALTx_10%D | 15.23% | 13.37% | 8.18% |
| Emp_MALTx_50%D | 14.55% | 12.77% | 7.80% |
| Emp_MALTx_20% | 15.04% | 13.22% | 8.06% |
