# Supplementary material for: Assessing alignment-based taxonomic classification of ancient microbial DNA
Source: PeerJ. 2019 Mar 13;7:e6594. doi: 10.7717/peerj.6594 (PMC6420809; doi:10.7717/peerj.6594)
Supplement: Supplemental Information 22 [file peerj-07-6594-s022.docx]

| **2014nr-specific** | **2017nt-specific** | **HOMD-specific** | **RefSeqGCS-specific** |
| --- | --- | --- | --- |
| Candidatus Koribacter versatilis | uncultured bacterium | Bacteroides pyogenes | Porphyromonas sp. KLE 1280 |
| Parabacteroides merdae | Bacteroides cellulosilyticus | Prevotella shahii | Prevotella conceptionensis |
| Capnocytophaga sp. CM59 | Bacteroides fragilis | Capnocytophaga sp. oral taxon 336 | Prevotella sp. HMSC073D09 |
| Capnocytophaga sp. oral taxon 324 | Bacteroides salanitronis | Capnocytophaga sputigena | Phocaeicola abscessus |
| Riemerella anatipestifer | Bacteroides thetaiotaomicron | Bacteroidetes bacterium oral taxon 274 | Leptotrichia trevisanii |
| Nitrospira defluvii | Barnesiella viscericola | Fusobacterium sp. oral taxon 370 | Desulfobulbus elongatus |
| Methylobacterium nodulans | Porphyromonas asaccharolytica | Bradyrhizobium elkanii | Desulfobulbus mediterraneus |
| Azorhizobium caulinodans | Prevotella dentalis | Achromobacter xylosoxidans | Acinetobacter venetianus |
| Neisseria sp. oral taxon 014 | Prevotella denticola | Delftia acidovorans | Actinomyces glycerinitolerans |
| Desulfomicrobium baculatum | Prevotella fusca | Desulfobulbus sp. oral taxon 041 | Actinomyces provencensis |
| Helicobacter pylori | Prevotella melaninogenica | Campylobacter rectus | Actinomyces slackii |
| Photorhabdus luminescens | Prevotella ruminicola | Aggregatibacter sp. oral taxon 458 | Actinomyces sp. HMSC035G02 |
| Pseudoxanthomonas suwonensis | Prevotella scopos | Haemophilus haemolyticus | Actinomyces sp. HPA0247 |
| Schlesneria paludicola | Prevotella sp. oral taxon 299 | Pseudomonas aeruginosa | Actinomyces sp. Marseille-P2825 |
| Treponema caldarium | Capnocytophaga canimorsus | Pseudomonas fluorescens | Sanguibacter keddieii |
| Treponema phagedenis | Capnocytophaga haemolytica | Stenotrophomonas maltophilia | Atopobium sp. HMSC064B08 |
| Actinomyces sp. ICM39 | Chryseobacterium indologenes | Actinomyces sp. oral taxon 877 | Eggerthellaceae bacterium AT8 |
| Actinomyces sp. ICM58 | Fusobacterium hwasookii | Parascardovia denticolens | Gemella cuniculi |
| Actinomyces sp. oral taxon 848 | Ramlibacter tataouinensis | Arsenicicoccus sp. oral taxon 190 | Abiotrophia sp. HMSC24B09 |
| Actinomyces turicensis | Variovorax sp. PAMC 28711 | Propionibacterium acidifaciens | Enterococcus faecalis |
| Actinomyces viscosus | Neisseria meningitidis | Olsenella profusa | Enterococcus faecium |
| Bifidobacterium thermophilum | Enterobacter cloacae | Chloroflexi bacterium oral taxon 439 | Pediococcus acidilactici |
| Corynebacterium durum | Haemophilus influenzae | Gemella bergeri | Streptococcus sp. DD04 |
| Brachybacterium faecium | Haemophilus parainfluenzae | Granulicatella adiacens | Clostridium sp. Marseille-P3244 |
| Cutibacterium acnes | Histophilus somni | Streptococcus infantis | Eubacterium callanderi |
| Cryptobacterium curtum | Gemmata sp. SH-PL17 | Streptococcus sinensis | Oribacterium asaccharolyticum |
| Slackia sp. CM382 | Treponema putidum | Mogibacterium timidum | Lachnospiraceae bacterium 1_1_57FAA |
| Anaerolinea thermophila | Actinomyces hongkongensis | Johnsonella ignava | Peptoanaerobacter stomatis |
| Bacillus coagulans | Actinomyces radingae | Oribacterium sp. oral taxon 108 | Peptostreptococcaceae bacterium oral taxon 113 |
| Gemella haemolysans | Actinomyces sp. Chiba101 | Selenomonas sp. oral taxon 137 | Ruminococcus flavefaciens |
| Streptococcus mutans | Actinomyces sp. Marseille-P2985 | Selenomonas sp. oral taxon 138 | Intestinimonas massiliensis |
| Streptococcus sp. 2_1_36FAA | Actinomyces sp. pika_114 | Selenomonas sp. oral taxon 892 | Pseudoflavonifractor sp. Marseille-P3106 |
| Streptococcus sp. oral taxon 056 | Actinomyces succiniciruminis | Veillonella sp. AS16 | Thermoanaerobacter siderophilus |
| Clostridium sp. BNL1100 | Corynebacterium aquilae | Veillonella sp. oral taxon 158 | Eggerthia catenaformis |
| Mogibacterium sp. CM50 | Corynebacterium diphtheriae | Parvimonas sp. oral taxon 110 | Methanobrevibacter arboriphilus |
| Marvinbryantia formatexigens | Corynebacterium glutamicum | Peptoniphilus sp. oral taxon 836 | Methanobrevibacter wolinii |
| Oribacterium sinus | Corynebacterium mustelae |  |  |
| Roseburia intestinalis | Corynebacterium pseudotuberculosis |  |  |
| Roseburia inulinivorans | Corynebacterium ulcerans |  |  |
| Ruminococcus sp. SR1/5 | Corynebacterium vitaeruminis |  |  |
| Syntrophothermus lipocalidus | Gordonia polyisoprenivorans |  |  |
| Pseudoflavonifractor capillosus | Lawsonella clevelandensis |  |  |
| Faecalitalea cylindroides | Arthrobacter saudimassiliensis |  |  |
| Acetonema longum | Libanicoccus massiliensis |  |  |
| Pelosinus fermentans | Olsenella sp. Marseille-P2300 |  |  |
| Dialister succinatiphilus | Olsenella umbonata |  |  |
| candidate division TM7 genomosp. GTL1 | Eggerthella sp. YY7918 |  |  |
| candidate division TM7 single-cell isolate TM7a | Gordonibacter sp. Marseille-P2775 |  |  |
| candidate division TM7 single-cell isolate TM7c | Paenibacillus polymyxa |  |  |
| Methanobacterium paludis | Staphylococcus aureus |  |  |
| Methanobacterium sp. Maddingley MBC34 | Streptococcus dysgalactiae |  |  |
|  | Streptococcus marmotae |  |  |
|  | Streptococcus parasanguinis |  |  |
|  | Streptococcus pyogenes |  |  |
|  | Streptococcus sp. NPS 308 |  |  |
|  | Streptococcus sp. oral taxon 431 |  |  |
|  | Christensenella massiliensis |  |  |
|  | Eubacterium limosum |  |  |
|  | Lachnoclostridium phocaeense |  |  |
|  | Flavonifractor plautii |  |  |
|  | Selenomonas sp. oral taxon 136 |  |  |
|  | Selenomonas sp. oral taxon 478 |  |  |
|  | Dialister pneumosintes |  |  |
|  | Megasphaera elsdenii |  |  |
|  | Ndongobacter massiliensis |  |  |
|  | Methanosphaera stadtmanae |  |  |
|  | Methanothermus fervidus |  |  |
|  |  |  |  |
