# Supplementary material for: Assessing alignment-based taxonomic classification of ancient microbial DNA
Source: PeerJ. 2019 Mar 13;7:e6594. doi: 10.7717/peerj.6594 (PMC6420809; doi:10.7717/peerj.6594)
Supplement: Supplemental Information 23 [file peerj-07-6594-s023.docx]

| **Sample/database** | **Mean read length** | **# total reads** | **# reads assigned taxonomy** | **% total reads assigned taxonomy** | **% total reads unassigned** | **Fold-increase in number of reads assigned over 2014nr** |
| --- | --- | --- | --- | --- | --- | --- |
| A11105_AfrSudan1-2014nr | 41 | 5,246 | 98 | 1.9% | 98.1% |  |
| A11105_AfrSudan1-RefSeqGCS | 41 | 5,246 | 1,688 | 32.2% | 67.8% | 17.2 |
| A11106_AfrSudan2-2014nr | 34 | 81,155 | 202 | 0.2% | 99.8% |  |
| A11106_AfrSudan2-RefSeqGCS | 34 | 81,155 | 3,545 | 4.4% | 95.6% | 17.5 |
| A12014_EuroHG1-2014nr | 53 | 145,907 | 2,188 | 1.5% | 98.5% |  |
| A12014_EuroHG1-RefSeqGCS | 53 | 145,907 | 57,071 | 39.1% | 60.9% | 26.1 |
| A12017_EuroHG2-2014nr | 51 | 93,208 | 1,184 | 1.3% | 98.7% |  |
| A12017_EuroHG2-RefSeqGCS | 51 | 93,208 | 35,762 | 38.4% | 61.6% | 30.2 |
| A12824_LBK3-2014nr | 48 | 39,463 | 330 | 0.8% | 99.2% |  |
| A12824_LBK3-RefSeqGCS | 48 | 39,463 | 17,716 | 44.9% | 55.1% | 53.7 |
| A12826_LBK1-2014nr | 47 | 125,570 | 781 | 0.6% | 99.4% |  |
| A12826_LBK1-RefSeqGCS | 47 | 125,570 | 45,990 | 36.6% | 63.4% | 58.9 |
| A12829_LBK2-2014nr | 47 | 158,406 | 1,351 | 0.9% | 99.1% |  |
| A12829_LBK2-RefSeqGCS | 47 | 158,406 | 78,905 | 49.8% | 50.2% | 58.4 |
| A13204_AfrPP2-2014nr | 43 | 821,114 | 1,894 | 0.2% | 99.8% |  |
| A13204_AfrPP2-RefSeqGCS | 43 | 821,114 | 233,981 | 28.5% | 71.5% | 123.5 |
| A13209_AfrSF3-2014nr | 41 | 3,391,497 | 2,256 | 0.1% | 99.9% |  |
| A13209_AfrSF3-RefSeqGCS | 41 | 3,391,497 | 1,185,540 | 35.0% | 65.0% | 525.5 |
| A13210-AfrSF4-2014nr | 44 | 308,202 | 532 | 0.2% | 99.8% |  |
| A13210-AfrSF4-RefSeqGCS | 44 | 308,202 | 69,405 | 22.5% | 77.5% | 130.5 |
| A13213_AfrPP1-2014nr | 44 | 2,642,915 | 7,146 | 0.3% | 99.7% |  |
| A13213_AfrPP1-RefSeqGCS | 44 | 2,642,915 | 853,865 | 32.3% | 67.7% | 119.5 |
| A13232_IndRev1-2014nr | 49 | 116,542 | 2,892 | 2.5% | 97.5% |  |
| A13232_IndRev1-RefSeqGCS | 49 | 116,542 | 62,908 | 54.0% | 46.0% | 21.8 |
| A13234_IndRev2-2014nr | 44 | 11,464,274 | 85,625 | 0.7% | 99.3% |  |
| A13234_IndRev2-RefSeqGCS | 44 | 11,464,274 | 6,080,790 | 53.0% | 47.0% | 71.0 |
| A13344_ET11_EBC-2014nr | 30 | 238,422 | 65 | 0.03% | 100.0% |  |
| A13344_ET11_EBC-RefSeqGCS | 30 | 238,422 | 4,570 | 1.9% | 98.1% | 70.3 |
| A8812_JewBury1-2014nr | 51 | 51,926 | 875 | 1.7% | 98.3% |  |
| A8812_JewBury1-RefSeqGCS | 51 | 51,926 | 27,054 | 52.1% | 47.9% | 30.9 |
| A8824_JewBury2-2014nr | 50 | 71,125 | 929 | 1.3% | 98.7% |  |
| A8824_JewBury2-RefSeqGCS | 50 | 71,125 | 36,979 | 52.0% | 48.0% | 39.8 |
| AFR8-EBC-2014nr | 39 | 9,076 | 55 | 0.6% | 99.4% |  |
| AFR8-EBC-RefSeqGCS | 39 | 9,076 | 6,034 | 66.5% | 33.5% | 109.7 |
| CHIMP_2014nr | 57 | 17,575,167 | 1,266,014 | 7.2% | 92.8% |  |
| CHIMP-RefSeqGCS | 57 | 17,575,167 | 7,526,697 | 42.8% | 57.2% | 5.9 |
| ELSIDRON1-2014nr | 57 | 50,238,935 | 1,333,986 | 2.7% | 97.3% |  |
| ELSIDRON1-RefSeqGCS | 57 | 50,238,935 | 15,771,444 | 31.4% | 68.6% | 11.8 |
| ELSIDRON2-2014nr | 60 | 48,231,792 | 3,171,401 | 6.6% | 93.4% |  |
| ELSIDRON2-RefSeqGCS | 60 | 48,231,792 | 19,216,338 | 39.8% | 60.2% | 6.1 |
| Modern-2014nr | 67 | 29,469,839 | 6,991,350 | 23.7% | 76.3% |  |
| Modern-RefSeqGCS | 67 | 29,469,839 | 23,754,716 | 80.6% | 19.4% | 3.4 |
| SPYII-2014nr | 61 | 4,041,681 | 324,843 | 8.0% | 92.0% |  |
| SPYII-RefSeqGCS | 61 | 4,041,681 | 1,649,560 | 40.8% | 59.2% | 5.1 |
| War-B61-Med.Germany-2014nr | 82 | 13,260,566 | 4,544,233 | 34.3% | 65.7% |  |
| War-B61-Med.Germany-RefSeqGCS | 82 | 13,260,566 | 8,196,949 | 61.8% | 38.2% | 1.8 |
| War-G12-Med.Germany-2014nr | 88 | 8,999,409 | 3,754,002 | 41.7% | 58.3% |  |
| War-G12-Med.Germany-RefSeqGCS | 88 | 8,999,409 | 5,693,022 | 63.3% | 36.7% | 1.5 |
|  |  |  |  |  |  |  |
| **AVERAGE 2014nr** | 51 | 7,982,560 | 895,593 | 5.8% | 94.2% |  |
| **AVERAGE RefSeqGCS** | 51 | 7,982,560 | 3,775,439 | 41.8% | 58.2% | **64.2** |
