# Supplementary material for: Assessing alignment-based taxonomic classification of ancient microbial DNA
Source: PeerJ. 2019 Mar 13;7:e6594. doi: 10.7717/peerj.6594 (PMC6420809; doi:10.7717/peerj.6594)
Supplement: Supplemental Information 24 — Species-level taxonomic composition of extraction blank control samples that were subtractively removed (filtered) from the ancient dental calculus samples. [file peerj-07-6594-s024.docx]

| **Taxon** | **A13344_ET11_EBC-2014nr** | **AFR8-EBC-2014nr** | **A13344_ET11_EBC-RefSeqGCS** | **AFR8-EBC-RefSeqGCS** |
| --- | --- | --- | --- | --- |
| Pedobacter cryoconitis | 0 | 0 | 54 | 88 |
| Rhodoplanes sp. Z2-YC6860 | 0 | 0 | 9 | 0 |
| Porphyrobacter sp. CACIAM 03H1 | 0 | 0 | 12 | 0 |
| Paraburkholderia dilworthii | 0 | 0 | 55 | 0 |
| Ralstonia solanacearum | 0 | 1 | 35 | 0 |
| Comamonas testosteroni | 0 | 0 | 10 | 0 |
| Pluralibacter gergoviae | 0 | 0 | 29 | 0 |
| Halomonas chromatireducens | 0 | 0 | 11 | 0 |
| Acinetobacter baumannii | 0 | 3 | 0 | 0 |
| Acinetobacter junii | 0 | 0 | 0 | 183 |
| Acinetobacter sp. ATCC 27244 | 0 | 1 | 0 | 0 |
| Acinetobacter sp. CIP 102637 | 0 | 0 | 0 | 10 |
| Acinetobacter sp. NBRC 100985 | 0 | 1 | 0 | 0 |
| Acinetobacter sp. NIPH 284 | 0 | 0 | 0 | 112 |
| Acinetobacter venetianus | 0 | 0 | 0 | 79 |
| Pseudomonas alcaligenes | 0 | 0 | 65 | 0 |
| Pseudomonas fluorescens | 0 | 0 | 7 | 0 |
| Pseudomonas tolaasii | 0 | 0 | 17 | 0 |
| Pseudomonas sp. HMSC08G10 | 0 | 0 | 0 | 7 |
| Pseudomonas syringae | 0 | 0 | 0 | 53 |
| Mycobacterium sp. URHD0025 | 0 | 0 | 15 | 0 |
| Mycobacterium bovis | 0 | 0 | 0 | 375 |
| Brachybacterium muris | 0 | 0 | 0 | 13 |
| Nocardioides alkalitolerans | 0 | 0 | 9 | 0 |
| Nocardioides sp. CF8 | 0 | 0 | 4 | 0 |
| Streptomyces avermitilis | 0 | 0 | 52 | 0 |
| Streptomyces sp. TP-A0356 | 0 | 0 | 7 | 0 |
| Bacillus thuringiensis | 0 | 0 | 4 | 0 |
| Staphylococcus epidermidis | 0 | 0 | 1896 | 491 |
| Staphylococcus hominis | 26 | 0 | 0 | 0 |
| Enterococcus faecalis | 0 | 0 | 132 | 98 |
| Enterococcus faecium | 0 | 0 | 128 | 3825 |
| Pediococcus acidilactici | 0 | 0 | 9 | 0 |
